# 

**Published:** 1946

**Authors:** 


### CONTENTS.

| | Page |
|---|---|
| Nature and Science. By Hugh H. Carleton, M.A., D.M., B.Ch., F.R.C.P. | 1 |
| The Diagnosis and Treatment of Rheumatoid Arthritis. By G. D. Kersley, M.D., F.R.C.P. | 11 |
| The Management of Peptic Ulcer. By Norman C. Tanner, M.B., Ch.B., F.R.C.S. | 16 |
| The British Red Cross in North-West Europe. By Lieut.-Col. K. M. Agnew, D.S.O., M.C., R.A. (retired). | 31 |
| Reviews of Books | 43 |
| Editorial Notes | 47 |
| Meetings of Societies | 48 |
| The Medical Library of the University of Bristol | 50 |
| Publications Received | 54 |
| Local Medical Notes | 55 |

